# Antioxidant Capacities and Total Phenolic Contents Increase with Gamma Irradiation in Two Types of Malaysian Honey

**DOI:** 10.3390/molecules16086378

**Published:** 2011-07-27

**Authors:** Saba Zuhair Hussein, Kamaruddin Mohd Yusoff, Suzana Makpol, Yasmin Anum Mohd Yusof

**Affiliations:** 1 Department of Biochemistry, Faculty of Medicine, University Kebangsaan Malaysia, 50300 Kuala Lumpur, Malaysia; Email: sa78ba@yahoo.com (S.Z.H.); suzanamakpol@yahoo.com (S.M.); 2 Department of Molecular Medicine, Faculty of Medicine, University of Malaya, 50603 Kuala Lumpur, Malaysia; Email: kamaruddin77@um.edu.my

**Keywords:** Gelam honey, Nenas honey, antioxidant activity, phenolics, gamma irradiation

## Abstract

Two types of monofloral Malaysian honey (Gelam and Nenas) were analyzed to determine their antioxidant activities and total phenolic and flavonoid contents, with and without gamma irradiation. Our results showed that both types of honey can scavenge free radicals and exhibit high antioxidant-reducing power; however, Gelam honey exhibited higher antioxidant activity (p < 0.05) than Nenas honey, which is in good correlation (r = 0.9899) with its phenolic contents. Interestingly, we also noted that both irradiated honeys have higher antioxidant activities and total phenolic and flavonoid contents compared to nonirradiated honeys by Folin-Ciocalteu and UV-spectrophotometry methods, respectively. However, HPLC analysis for phenolic compounds showed insignificant increase between irradiated and nonirradiated honeys. The phenolic compounds such as: caffeic acid, chlorogenic acid, ellagic acid, *p*- coumaric acid, quercetin and hesperetin as indicated by HPLC method were found to be higher in Gelam honey versus Nenas honey. In conclusion, irradiation of honey causes enhanced antioxidant activities and flavonoid compounds.

## 1. Introduction

Honey is a collection of nectar from many plants processed by honey bees. This natural product is well known for its high nutritional and prophylactic medicinal value. Apitherapy (the medical use of honey bee products) has recently become the focus of attention as a form of folk and preventive medicine for treating certain conditions and diseases, as well as promoting overall health and well being [1]. It has been reported to be effective in gastrointestinal disorders [2], in the healing of wounds and burns [3], as an antimicrobial or antibacterial agent [4] and to provide gastric protection against acute and chronic gastric lesions [5]. However, because some of these diseases are a consequence of oxidative damage, it seems that part of the therapeutic properties of honey is due to it antioxidant capacity [6]. The term “oxidative stress” describes the lack of equilibrium between the free radicals generated and the antioxidant protective activity in a given organism. Antioxidative protection against oxidation is thought to prevent some chronic diseases such as cardiovascular disease, stroke, cancer, chronic respiratory diseases and diabetes [7,8]. 

Many authors have demonstrated that honey serves as a source of natural antioxidants [4,9,10] and honey has been shown to reduce the risk of heart disease, cancer, cataracts and inflammatory processes [11,12]. The components in honey that are responsible for its antioxidative effect are the phenolics. It was reported that the antioxidant capacity and composition of honey depend on the floral source, seasonal and environmental factors, as well as the method of processing honey [13].

Honey has two sources of contamination with microorganisms: primary sources include pollen, the digestive tracts of honey bees, dust, air, soil and nectar; secondary sources are those arising from honey manipulation by people, they include air, food handlers, cross-contamination, equipment and buildings [14,15]. Gamma irradiation as a phytosanitary treatment has been proven to be safe and effective in improving the hygienic quality of various foods and herbal materials [16,17]. According to Molan and Allen [18] 25 kGy of gamma-irradiation is sufficient to achieve sterility for honey. 

To our knowledge, no studies have shown the effect of gamma irradiation on the antioxidant capacity and phenolic compounds of honey. The aim of this study was to evaluate and compare the antioxidant properties, flavanoid and phenolic contents of Malaysian honey using two different solvents (distilled water and methanol). Our secondary aim was to evaluate the effect of irradiation on the antioxidative properties and phenolic components of Gelam and Nenas honey.

## 2. Results and Discussion

### 2.1. Ferric reducing/antioxidant power (FRAP) assay

The FRAP assay measures the reducing potential of an antioxidant that reacts with a ferric tripyridyltriazine (Fe^3+^ - TPTZ) complex to produce a colored ferrous tripyridyltriazine (Fe^2+^ - TPTZ) [19,20]. Generally, the reducing properties are associated with the presence of compounds, which exert their action by breaking the free radical chair through the donation of a hydrogen atom [21,22]. The FRAP values of the two types of Malaysian honey dissolved in water or methanol in the concentration range of 0.1–0.4 g/mL are shown in (Table 1). In general, an increase in the concentration of honey resulted in significant (p < 0.05) increases in the ferric reducing antioxidant power (FRAP) for all honey samples. This result is similar to the study of Malaysian herbs performed by Huda-Faujan *et al*. [23], who found that all of the herbs extracts showed increased reducing ability as the concentration of extracts were increased. 

**Table 1:** Ferric Reducing Antioxidant Power (FRAP) values of Gelam and Nenas honeys before and after irradiation.

Concentration (g/mL)	Honey Dissolved in Distilled water						
	NNI		NI		GNI		GI
0.1	105.64 ± 2.03 ^a,c^		200.74 ± 2.51 ^b,c^		210.08 ± 2.68 ^a,d^		283.24 ± 8.44 ^b,d^
0.2	179.19 ± 5.19 ^a,c^		354.07 ± 9.36 ^b,c^		367.37 ± 13.1 ^a,d^		571.13 ± 1.64 ^b,d^
0.3	255.37 ± 4.56 ^a,c^		513.40 ± 8.32 ^b,c^		526.80 ± 12.0 ^a,d^		909.27 ± 16.8 ^b,d^
0.4	311.4 ± 7.97 ^a,c^		660.20 ± 68.5 ^b,c^		689.37 ± 23.6 ^a,d^		1108.9 ± 28.5 ^b,d^
**Concentration (g/mL)**	**Honey Dissolved in Methanol**						
	**NNI**		**NI**		**GNI**		**GI**	
0.1	79.07 ± 0.81 ^a,c^		182.93 ± 6.05 ^b,c^		188.97 ± 5.44 ^a,d^		270.70 ± 13.8 ^b,d^
0.2	147.83 ± 0.60 ^a,c^		309.60 ± 15.8 ^b,c^		310.72 ± 11.6 ^a,d^		587.60 ± 40.7 ^b,d^
0.3	242.50 ± 5.68 ^a,c^		458.70 ± 22.7 ^b,c^		508.77 ± 39.0 ^a,d^		868.03 ± 14.5 ^b,d^
0.4	283.30 ± 21.9 ^a,c^		546.50 ± 34.6 ^b,c^		618.30 ± 26.1 ^a,d^		1091.6 ± 44.9 ^b,d^

GI, Gelam irradiated; GNI, Gelam nonirradiated; NI, Nenas irradiated; NNI, Nenas nonirradiated honey. Data are expressed as the mean ± standard deviation from three independent experiments (n = 3). Superscripts **a** and **b** indicate significant differences (P < 0.05) between irradiated and nonirradiated (similar honey type) at similar concentrations and solvent conditions. Superscripts **c** and **d** indicate significant difference (P < 0.05) between irradiated or nonirradiated (different honey type) at similar concentrations and solvent conditions.

When comparing the FRAP values between different solvents, we found that both methanol and water gave similar antioxidant reducing power for both types of honey. Generally, the FRAP value of the Gelam honey was found to be significantly (p < 0.05) higher than that of the Nenas honey in all concentrations and solvents. Aljadi *et al*. [10] also reported that Gelam honey has a significantly higher FRAP value than coconut honey. We also observed that both irradiated Gelam and Nenas honeys indicated a significantly (p < 0.05) higher FRAP value compared to their non-irradiated counterparts in both solvents. Gamma irradiation is a method of decontamination for food and herbal materials [16,17]. It has been used to prevent microbial and bacterial contamination in honey. Molan *et al*. [18] and Postmes *et al*. [24] reported that irradiation rendered the honey sterile without affecting its antibacterial activity. 

To our knowledge, no studies have reported on the effect of gamma-irradiation on the antioxidant properties of honey. Song *et al*. [25] reported that the antioxidant capacity of irradiated carrot juice was higher than that of the non-irradiated juice; while Stajner *et al*. [26] found higher antioxidant capacities in irradiated versus non-irradiated soya. The increase in antioxidant activity following irradiation might be due to the degradation of some high molecular weight components, and changing the solubility of these compounds in the test solvents gave rise to more phenolic compounds [27].

### 2.2. The free radical-scavenging activity

One of the mechanisms to investigate antioxidant activity is to study the scavenging effect on proton radicals. In the present study, investigation of the total antioxidant capacity was measured as the cumulative capacity of the compounds in the sample that can scavenge free radicals using the DPPH reaction. The presence of antioxidants in the sample leads to the disappearance of DPPH radical chromogens, which can be detected spectrophotometrically at 517 nm [28].

The radical scavenging activities of the honey samples were analyzed in water and methanol solvents using 1,1-diphenyl-2-picrylhydrazyl radicals (DPPH). For both types of honey, the scavenging activity was found to increase significantly (p < 0.05) with increasing concentrations in both solvents (Table 2). As seen in Table 2 both types of honey Gelam and Nenas (irradiated and nonirradiated) have high scavenging activities in both solvents. Gelam honey, dissolved in both solvents and at all concentrations, has a significantly (p < 0.05) higher ability to scavenge the free radical compared to Nenas honey. 

**Table 2:** Radical scavenging activity (% inhibition) of Gelam and Nenas honeys before and after irradiation.

Concentration (g/mL)	Honey dissolved in distilled water			
	NNI	NI	GNI	GI
0.1	3.69 ± 0.07 ^a,c^	18.00 ± 0.21 ^b,c^	31.46 ± 0.36 ^a,d,e^	54.60 ± 1.82 ^b,d,e^
0.2	6.24 ± 3.25 ^a,c^	28.63 ± 5.98 ^b,c^	53.58 ± 1.57 ^a,d,e^	73.13 ± 1.08 ^b,d,e^
0.3	9.07 ± 1.25 ^a,c,e^	35.71 ± 0.37 ^b,c^	69.39 ± 0.74 ^a,d,e^	77.93 ± 0.49 ^b,d,e^
0.4	28.67 ± 0.95 ^a,c,e^	52.79 ± 0.82 ^b,c^	76.29 ± 0.58 ^a,d,e^	82.68 ± 0.80 ^b,d,e^
**Concentration (g/mL)**	**Honey dissolved in methanol**			
	**NNI**	**NI**	**GNI**	**GI**
0.1	1.66 ± 1.12 ^a,c^	16.61 ± 1.85 ^b,c^	24.37 ± 3.98 ^a,d,f^	51.51 ± 0.42 ^b,d,f^
0.2	3.75 ± 1.26 ^a,c^	25.96 ± 4.98 ^b,c^	42.61 ± 2.40 ^a,d,f^	64.44 ± 0.14 ^b,d,f^
0.3	5.11 ± 1.86 ^a,c,f^	33.97 ± 1.31 ^b,c^	62.33 ± 4.15 ^a,d,f^	68.52 ± 1.77 ^b,d,f^
0.4	17.74 ± 1.33 ^a,c,f^	51.04 ± 0.22 ^b,c^	68.22 ± 0.94 ^a,d,f^	79.26 ± 0.14 ^b,d,f^

GI, Gelam irradiated; GNI, Gelam nonirradiated; NI, Nenas irradiated; NNI, Nenas nonirradiated honey. Data are expressed as the mean ± Standard Deviation from three independent experiments (n = 3). Superscripts **a** and **b** indicate significant differences (P < 0.05) between irradiated and nonirradiated (similar honey type) at similar concentrations and solvent conditions. Superscripts **c** and **d** indicate significant differences (P < 0.05) between irradiated or nonirradiated (different honey type) at similar concentrations and solvent conditions. Superscripts **e** and **f** indicate significant differences (P < 0.05) between distilled water and methanol solvents of the similar honey type and concentration.

Gamma-irradiation caused a significant (p < 0.05) increase in the free radical-scavenging activity for both Gelam and Nenas honeys at all concentrations and solvents tested. Many researchers [4,6,13,29,30,31,32,33,34] have demonstrated the high scavenging activity of honey by various assays such as DPPH, ABTS, ONOO^- ^and NBT; however, none of them have compared the antioxidant properties of honey between the irradiated and nonirradiated forms. We found reports on the effects of irradiation on the antioxidant properties of other food products besides honey. Jo *et al*. [35] reported that irradiation (10 and 20 kGy) on green tea extracts increased its antioxidant properties while Khattak *et al*. [36] and Stajner *et al*. [26] found that irradiation increased the antioxidant properties of the *Glycyrrhiza glabra* root and soybean, respectively. According to Khattak *et al*. [27], gamma irradiation enhanced the free radical scavenging activity in *Nigella sativa* seeds. On the contrary, Lampart-Szcrapa *et al*. [37] reported that increasing doses of irradiation decreased the antioxidant effects of lupin seed extracts.

### 2.3. Total Flavonoid contents (TFC)

The total flavonoid contents in Gelam and Nenas honey dissolved in methanol are shown in (Table 3). Total flavonoid contents increased significantly (p &lt; 0.05) with increasing honey concentrations.

**Table 3:** Flavonoid contents (mg Rutin equivalent/100 g) of Gelam and Nenas honeys before and after irradiation.

Concentration (g/mL)	NNI	NI	GNI	GI
0.1	1.23 ± 0.19 ^a^	1.96 ± 0.11 ^b,c^	1.47 ± 0.03 ^a^	2.93 ± 0.13 ^b,d^
0.2	1.86 ± 0.39 ^a,c^	2.89 ± 0.16 ^b,c^	3.38 ± 0.02 ^a,d^	5.05 ± 0.08 ^b,d^
0.3	3.79 ± 0.10 ^a,c^	4.23 ± 0.02 ^b,c^	4.24 ± 0.05 ^a,d^	5.68 ± 0.14 ^b,d^
0.4	4.52 ± 0.01 ^c^	4.79 ± 0.15 ^c^	4.94 ± 0.26 ^a,d^	6.92 ± 0.81 ^b,d^

GI, Gelam irradiated; GNI, Gelam nonirradiated; NI, Nenas irradiated; NNI, Nenas nonirradiated honey. Data are expressed as the mean ± standard deviation from three independent experiments (n = 3). Superscripts **a** and **b** indicate significant differences (P < 0.05) between irradiated and nonirradiated (similar honey type) at similar concentrations. Superscripts **c** and **d** indicate significant differences (P < 0.05) between irradiated or nonirradiated (different honey type) at similar concentrations.

Gelam honey has a significantly (p < 0.05) higher amount of flavonoids than Nenas honey. Similarly, we found that irradiated Gelam and Nenas honeys exhibited a significantly (p < 0.05) higher content of flavonoids than their nonirradiated counterparts. 

Previous studies have reported high flavonoid contents in different kinds of honey such as Portuguese, Burkina Fasan and Cuban honeys [6,29,38]. Flavonoids are recognized for their high pharmacological activities as radical scavengers [39]. Recent interest in these substances has been stimulated by the potential health benefits arising from their antioxidant activities and free radical scavenging capacities in coronary heart disease and cancer [40]. 

### 2.4. Total phenolic contents (TPC)

Table 4 shows the total phenolic contents of Gelam and Nenas honey. Total phenolic contents increased significantly with increasing honey concentrations for both Gelam and Nenas honeys in both solvents (water and methanol). There were no significant differences of total phenolic contents between water and methanol solvents for either Gelam or Nenas honey at any of the concentrations tested. However, Gelam honey exhibited significantly (p < 0.05) higher total phenolic contents than Nenas honey at all tested concentrations for both solvents. The total phenolic contents vary between different honey samples depending on the geographical location of the different floral sources, such as Malaysia, Burkina Faso, Turkey and Croatia [4,10,29,33,41].

**Table 4:** Total phenolic contents (mg Rutin equivalent/100 g) of Gelam and Nenas honeys before and after irradiation.

Concentration (g/mL)	Honey Dissolved in Distilled water			
	NNI	NI	GNI	GI
0.1	3.62 ± 0.18 ^a,c^	9.66 ± 0.32 ^b,c^	8.47 ± 0.20 ^a,d^	18.78 ± 1.60 ^b,d^
0.2	9.17 ± 1.00 ^a,c^	19.86 ± 0.90 ^b,c,e^	21.09 ± 0.37 ^a,d,e^	42.40 ± 0.34 ^b,d^
0.3	15.42 ± 0.35 ^a,c^	28.62 ± 1.27 ^b,c^	30.32 ± 0.77 ^a,d^	56.59 ± 1.24 ^b,d,e^
0.4	21.60 ± 0.45 ^a,c^	38.91 ± 1.64 ^b,c^	41.76 ± 0.84 ^a,d,e^	72.64 ± 0.89 ^b,d^
**Concentration (g/mL)**	**Honey Dissolved in Methanol**			
	**NNI**	**NI**	**GNI**	**GI**
0.1	3.43 ± 0.31 ^a,c^	10.0 ± 0.91 ^b,c^	9.44 ± 1.69 ^a,d^	19.72 ± 1.44 ^b,d^
0.2	8.65 ± 1.17 ^a,c^	18.11 ± 1.06 ^b,c,f^	22.67 ± 0.59 ^a,d,f^	41.80 ± 1.38 ^b,d^
0.3	15.26 ± 0.18 ^a,c^	29.13 ± 0.11 ^b,c^	29.39 ± 0.49 ^a,d^	51.79 ± 0.24 ^b,d,f^
0.4	21.33 ± 1.40 ^a,c^	37.98 ± 0.10 ^b,c^	35.99 ± 1.03 ^a,d,f^	71.51 ± 1.32 ^b,d^

GI, Gelam irradiated; GNI, Gelam nonirradiated; NI, Nenas irradiated; NNI, Nenas nonirradiated honey. Data are expressed as the mean ± standard deviation from three independent experiments (n = 3). Superscripts **a** and **b** indicate significant difference (P < 0.05) between irradiated and nonirradiated (similar honey type) at similar concentrations and solvent conditions. Superscripts **c** and **d** indicate significant difference (P < 0.05) between irradiated or nonirradiated (different honey type) at similar concentrations and solvent conditions. Superscripts **e** and **f** indicate significant differences (P < 0.05) between distilled water and methanol solvents of the similar honey type and concentration.

Total phenolic contents in the irradiated honey were higher (both Gelam and Nenas) when compared with the nonirradiated honey. This could be due to radiolysis of phenolics (eg. Gallic acid, Caffeic acid, *etc*.) in an aqueous solution that led to their efficient degradation to a hydroxylation effect [42]. The increase in phenolic contents in this study correlates well with previous studies in which the ability of gamma-irradiation to increase phenolic content was observed in fresh vegetable juice [25], soybean [26], almond skin extracts [43], and spices such as clove and nutmeg [44]. However; Kim *et al*. [45] found no significant increase in the total phenolic contents in irradiated cumin when compared to that of the nonirradiated cumin, and Ahn *et al*. [46] found that an increasing dose of gamma-irradiation significantly reduces the phenolic contents in cut Chinese cabbage.

### 2.5. Correlation between Total phenolic contents (TPC) and antioxidant activities

**Figure 1:**
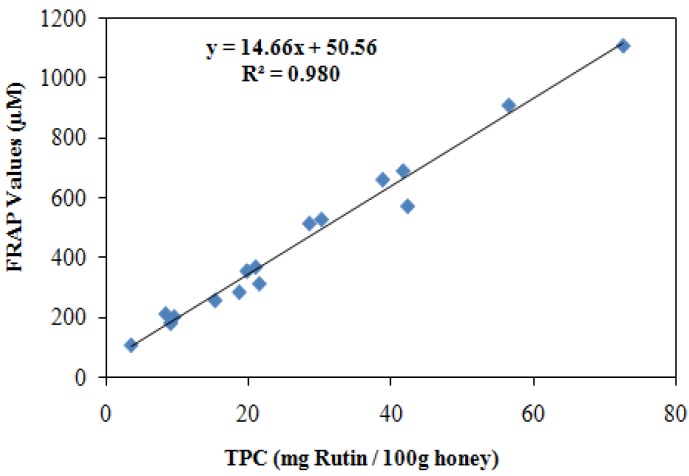
Correlation between the ferric reducing antioxidant power (FRAP value) of combined (Gelam and Nenas) honeys and the total phenolic contents (TPC) to obtain the r = 0.9899.

**Figure 2:**
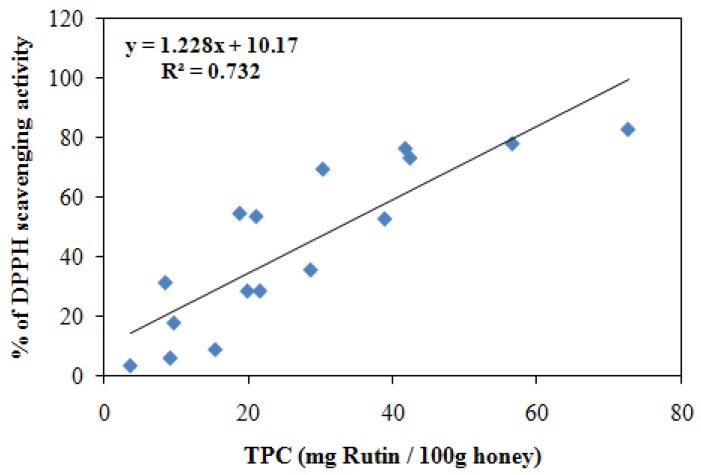
Correlation between the radical scavenging activity (% inhibition) using DPPH of combined (Gelam and Nenas) honey and total phenolic contents (TPC) to obtain the r = 0.855.

To analyze the correlation between total phenolic content and antioxidant activity, we plotted the values of antioxidant activities (FRAP & DPPH) with the total phenolic content of honey (Figure 1 and Figure 2). A significant linear correlation was found between FRAP and DPPH values of Gelam and Nenas honey with TPC (r = 0.9899 and r = 0.855, respectively), as well as between total flavonoid content and antioxidant activity (r = 0.917 by FRAP assay, and r = 0.785 by DPPH assay). Other studies have also found good correlations between antioxidant capacities and phenolic as well as flavonoid contents, indicating that the phenolics and flavonoids are one of the major components responsible for the antioxidant activity of honey [29,33,38,47,48]. A significant linear correlation between total flavonoid content and total phenolic content was observed in this study (r = 0.939) similar to findings of Socha *et al*. [34] who reported a significant linear correlation (r = 0.83) between total phenolic content and total flavonoid content in herb honeys. Table 5 summarizes the findings of other researchers in comparison with those of our study regarding the antioxidant capacity and total phenolic and flavonoid contents of different types of honey from different sources. Our Malaysian honeys, Gelam and Nenas have comparatively higher antioxidant reducing power compared to honey from Croatia, and Gelam honey has a higher radical scavenging activity by DPPH compared with commercial Indian honey. The total phenolic content of Gelam honey was almost similar to Croatian and Portuguese honey while the flavonoid content was very low compared to Portuguese honey. Additionally, Malaysian Tualang honey (obtained from deep forest) also has high antioxidant reducing power compared to Croatian honey.

**Table 5:** Antioxidant properties and total phenolic and flavonoid contents for different types of honey reported by some researchers for comparison with Malaysian honey.

Honey sources	Honey types	Antioxidant activity by FRAP assay (µM FeII)	Radical scavenging activity by DPPH (% inhibition)	Total phenolic contents (mg/100 g honey)	Total flavonoid contents (mg/100 g honey)
Croatian monofloral honey (Piljac-Zegarac *et al*., 2009) [41]	Jerusalem thorn	113.49 ± 2.91	-	48.58 ± 0.95	-
	Sunflower	113.81 ± 9.72	-	54.63 ± 1.20	-
	Sage	121.27 ± 4.70	-	55.40 ± 1.14	-
	Velebit winter	118.57 ± 4.54	-	44.43 ± 2.83	-
	Winter savory	99.68 ± 3.99	-	44.17 ± 2.24	-
	Amorpha	23.02 ± 2.79	-	25.66 ± 0.99	-
	Chestnut	84.60 ± 2.62	-	43.09 ± 2.68	-
	Linden	73.81 ± 6.75	-	40.88 ± 1.05	-
	Acasia	12.06 ± 1.98	-	21.61 ± 0.63	-
	Oilseed rape	52.22 ± 6.14	-	36.92 ± 2.53	-
	Goldenrod	92.86 ± 1.65	-	49.24 ± 2.02	-
Northeast Portugal honey (Ferreira *et al*., 2009) [6]	Light	-	-	22.61 ± 0.02	12.36 ± 0.01
	Amber	-	-	40.62 ± 1.72	34.27 ± 0.17
	Dark	-	-	72.77 ± 0.02	58.74 ± 0.04
Commercial Indian honey (Saxena *et al*., 2010) [32]	I	-	64 ± 0.7	98 ± 1.2	-
	II	-	59 ± 0.5	47 ± 0.2	-
	III	-	61 ± 0.9	83 ± 1.1	-
	IV	-	44 ± 0.6	67 ± 0.8	-
	V	-	67 ± 1.1	91 ± 1.4	-
	VI	-	71 ± 1.3	94 ± 0.8	-
	VII	-	48 ± 0.8	99 ± 1.3	-
Malaysian Honey (Mohamed *et al.*, 2010); Saba *et al*., 2010) [30,49]	Tualang	322.7 ± 1.7	41.3 ± 0.78	25.17 ± 0.79	-
	Gelam GNI (0.4 g/mL)	689.37 ± 23.6	76.29 ± 0.58	41.76 ± 0.84	2.64 ± 0.12
	Nenas NNI (0.4 g/mL)	311.4 ± 7.97	28.67 ± 0.95	21.60 ± 0.45	1.97 ± 0.21

### 2.6. Identification and quantification of phenolic compounds in Malaysian honey by HPLC

**Figure 3:**
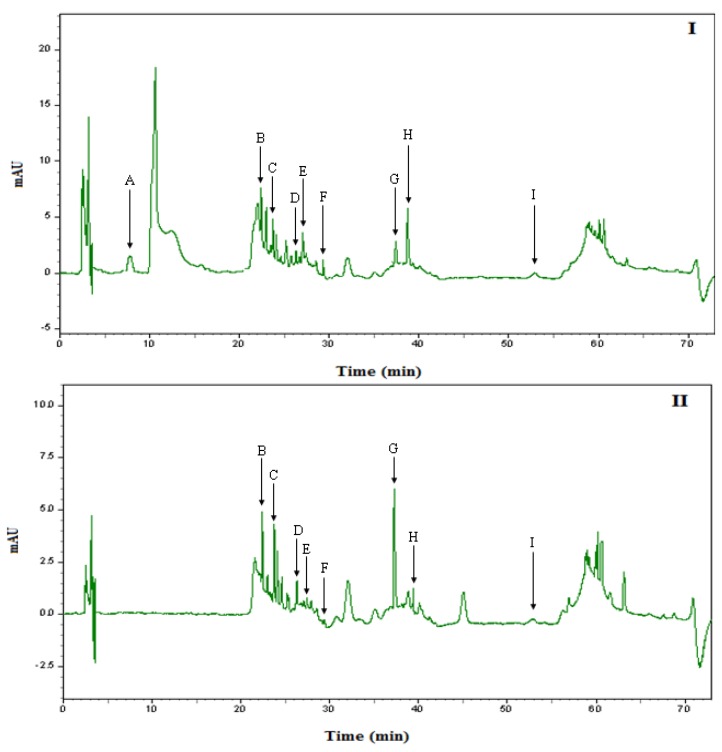
Chromatogram of Phenolic acid and Flavonoids detected in Malaysian Gelam honey using HPLC-UV absorption at (I) 290 nm and (II) 340 nm. A = gallic acid, B = chlorogenic acid, C = caffeic acid, D = *p*-coumaric acid, E = ferulic acid, F = ellagic acid, G = quercetin, H = hesperetin, I = chrysin.

**Figure 4:**
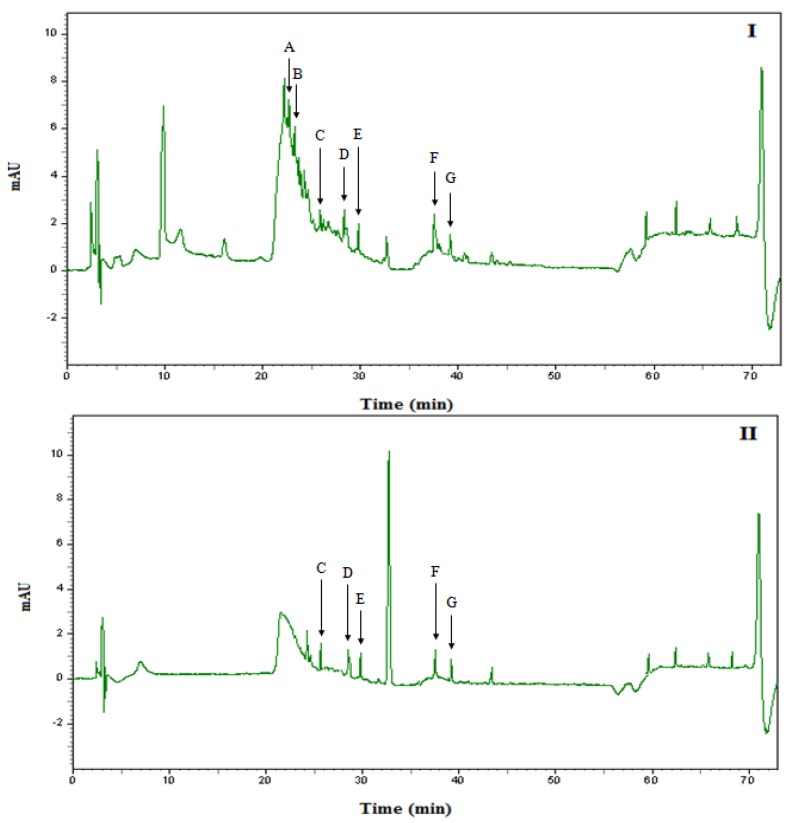
Chromatogram of Phenolic acid and Flavonoids detected in Malaysian Nenas honey using HPLC-UV absorption at (I) 290 nm and (II) 340 nm. A = chlorogenic acid, B = caffeic acid, C = *p*-coumaric acid, D = rutin, E = ellagic acid, F = quercetin, G = hesperetin.

Solid phase extraction (SPE), using C_18_ cartridges, were used to extract and recover phenolic compounds from honey. The recoveries were good for all standard phenolic compounds eluted from SPE, at 290 nm for phenol acids and 340 nm for flavonoids. The recoveries of phenolic acid standards were 71.5–98.8% while the flavonoid standards were 71.94–90.74%, indicating the suitability of this procedure for the recovery of phenolics in honey [50]. Figure 3 and Figure 4 show the UV absorption chromatograms of the two types of Malaysian honey (Gelam and Nenas) isolated by SPE at 290 nm and 340 nm. The concentrations of phenolic compounds in Malaysian honey are summarized in Table 6. The chromatograms of the extract samples from Malaysian honey showed a number of phenolic acids which absorb more strongly at 290 nm and flavonoids which absorb strongest at 340 nm [51]. Caffeic acid, chlorogenic acid, *p*-coumaric acid, ellagic acid, quercetin and hesperetin were identified in both types of honey. On the other hand, gallic acid, ferulic acid and chrysin were identified in Gelam honey while rutin was identified only in Nenas honey. Generally, Gelam honey contains significantly higher quantity of phenolic compounds than Nenas honey as calculated from the peak areas. However, there is no significant difference between both irradiated Gelam and Nenas honeys compared to non-irradiated honey. There is no information available on the effect of radiation on the phenolic compounds in honey. However; for some plant materials, diverse effects of radiation have been reported on total phenolic contents. Lee *et al*. [52] found increased total phenolic contents in tamarind juice, while Koseki *et al*. [53] reported significant decreased phenolic contents in dehydrated rosemary after irradiation at doses between 10–30 kGy. The difference in the effect of irradiation on total phenolic content may be due to plant type, geographical, environmental condition, phenolic content composition, temperature, extraction solvent, extraction procedure, and dose of gamma irradiation [36]. 

**Table 6:** Concentration of phenolic compounds detected in Gelam and Nenas honeys before and after irradiation by HPLC.

Phenolic Compounds	NNI		NI	
	Retention time (min)	µg/100 g honey at 290/340 nm	Retention time (min)	µg/100 g honey at 290/340 nm
Gallic acid	ND	ND	ND	ND
Chlorogenic acid	22.69	392.92 ± 42.22	22.69	433.73 ± 48.17
Caffeic acid	23.65	255.84 ± 11.83	23.66	278.26 ± 30.42
*P*- Coumaric caid	26.16	267.49 ± 13.99	26.18	312.10 ± 45.79
Ferulic caid	ND	ND	ND	ND
Rutin	28.52	1542.1 ± 60.21	28.50	1597.5 ± 125.37
Ellagic acid	29.73	306.33 ± 15.41	29.71	339.61 ± 44.41
Quercetin	37.75	1621.9 ± 91.11	37.76	1700.9 ± 93.97
Hesperetin	39.13	1493.9 ± 51.73	39.20	1536.6 ± 76.38
Chrysin	ND	ND	ND	ND
**Phenolic** ** Compounds**	**GNI**		**GI**	
	**Retention time (min)**	**µg/100 g honey at 290/340 nm**	**Retention time (min)**	**µg/100 g honey at 290/340 nm**
Gallic acid	7.86	859.43 ± 15.14	7.56	876.80 ± 7.47
Chlorogenic acid	22.40	502.77 ± 27.98	22.40	528.08 ± 6.31
Caffeic acid	23.73	428.84 ± 41.14	23.77	442.01 ± 32.70
*P*- Coumaric caid	26.19	301.45 ± 7.06	26.20	308.31 ± 18.69
Ferulic caid	26.91	356.93 ± 21.99	26.85	381.37 ± 17.07
Rutin	ND	ND	ND	ND
Ellagic acid	29.48	558.78 ± 36.68	29.50	575.67 ± 17.66
Quercetin	37.50	1588.9 ± 31.51	37.35	1594.30 ± 38.40
Hesperetin	39.20	1475.2 ± 5.40	39.21	1477.78 ± 1.91
Chrysin	53.22	1498.6 ± 3.50	53.31	1504.6 ± 3.20

GI, Gelam irradiated; GNI, Gelam nonirradiated; NI, Nenas irradiated; NNI, Nenas nonirradiated honey. Data are expressed as the mean ± Standard Deviation from three independent experiments (n = 3). The honey extract were analyzed with HPLC with UV detector set as 290/340 nm. The concentrations of phenolic compounds in honey extract were derived by calculating the peak area from the calibration curves of the standards used. ND = Not Detected.

Interestingly we also found unknown compounds in both types of honey, while some of them were present in higher concentrations when determined at 290 nm and 340 nm. Most of these unknown compounds are probably phenolic acids since their absorption was found mainly at 290 nm where phenolic acids absorb maximally [50,51]. Gelam honey exhibited higher unknown phenolic compounds than Nenas honey, in both irradiated and non-irradiated honeys. Irradiation exerts its effects as direct and indirect mechanisms; in case of indirect mechanism, radiolysis of water results in the production of radicals such as hydrated electrons, hydroxyl radicals and hydrogen atoms [54]. These radicals may break the glycosidic bonds that are present in honey, leading to the formation of new compounds. The increase in phenolic compounds in both gamma-irradiated honeys could be attributed to the release of phenolic compounds from glycosidic components and the degradation of the larger phenolic compounds into smaller ones by gamma irradiation [43].

## 3. Experimental

### 3.1. Chemicals and instruments

All the chemicals and reagents used were of analytical grade. The Folin-Ciocalteu reagent, sodium acetate, FeCl_3_.6H2O and 1,1-diphenyl-2-picrylhydrazyl (DPPH) were supplied by Sigma Chemicals Co. (USA). Methanol and sodium bicarbonate were obtained from Merck (Germany). Aluminum chloride and FeSO_4_.7H2O were from Fisher Scientific (UK). Rutin was obtained from Acros Organics (USA) and Tripyridyltriazine (TPTZ) from Fluka (Switzerland). Spectrophotometric measurements were performed on a double-beam UV-VIS spectrophotometer UV-160A (Shimadzu Corporation, Japan), High-Performance Liquid Chromatography (HPLC) 10A Shimadzu, Japan. 

### 3.2. Honey samples

Two of the most common monofloral Malaysian honeys were used in this study. Their floral sources were from *Melaleuca spp.* (Gelam) trees and *Ananas Comosus spp*. (Nenas) trees, and the honeys were named according to their floral sources. All the honeys were supplied by the Department of Agriculture in Malaysia (2009).

### 3.3. Gamma irradiation

The honey samples were irradiated in a cobalt-60 irradiator at 25 kGy [18] at the Malaysian Nuclear Agency in Selangor, Malaysia. Basically, honey samples were sealed properly and placed in a carton box. Two dosimeters were placed opposite the box. Gamma irradiation from Cobalt-60 source was passed through honey samples at 25 kGy, at the Malaysian Nuclear Agency, Selangor, Malaysia. Aluminium totes (JR 10000 IR 29 Tote Irradiator System, Canada) automatically enters and leaves the radiation room on roller conveyor system. Each tote measures 154 cm (depth) × 95 cm (length) and 63.5 cm (width). The tote encircles the Cobalt-60 source at a speed of 1 round/min/5 kGy amounting to 5 circles to produce 25 kGy. Once the dosage has been reached, the totes left the room and dosimeters were collected from dose mapping activity analysis to confirm dose requested. 

### 3.4. Ferric reducing / antioxidant power (FRAP) assay

The assay was carried out according to the methods described by Benzie and Strain [19], which is based on the reduction of Fe^3+^ - TPTZ to a blue colored Fe^2+^ FRAP reagent was freshly prepared by mixing 300 mM of acetate buffer (pH 3.6), 10 mM of TPTZ and 20 mM FeCl_3_.6H_2_O in a ratio of 10:1:1 at 37 °C. One hundred microliters of honey sample (0.1–0.4 g/mL) in distilled water or methanol and distilled water (300 µL) were added to FRAP reagent (3 mL) in a test tube. After four minutes of incubation at 37 °C, the absorbance was measured at 593 nm. The antioxidant potential of the sample was determined from a standard curve using FeSO_4_.7H_2_O at a concentration range between 100 and 3000 µM. 

### 3.5. The free radical-scavenging activity

The scavenging activity of honey samples for the radical 1,1-diphenyl-2-picrylhydrazyl (DPPH) was measured as described by Aljadi *et al*. [10], with some modifications. In the presence of an antioxidant, the purple color of DPPH decays, and the change of absorbency can be followed spectrophotometrically at 517 nm. Briefly, honey solution (0.75 mL, 0.1–0.4 g/mL) in distilled water or methanol was mixed with a 0.09 mg/mL solution of DPPH in methanol (1.5 mL). The mixture was left for 30 minutes at room temperature in the dark and the absorbance at 517 nm was measured. Antiradical activity (%) of the sample was calculated according to the formula:

Antiradical activity (%) = [(Ac – As) / Ac] × 100

where **Ac** is the absorbance of the control and **As** is the absorbance of the sample.

### 3.6. Total Flavonoid contents (TFC)

The total flavonoid content was determined spectrophotometrically according to Djeridane *et al*. [55]. This method is based on the formation of a flavonoid-aluminum complex with maximum absorptivity at 430 nm. Rutin was used to make the calibration curve. One milliliter of honey sample (0.1–0.4 g/mL) in distilled water or methanol was mixed with 2% aluminum chloride in methanol (1 mL). Following incubation for 15 minutes at room temperature, the absorbance of the mixture was measured at 430 nm with a UV-Vis spectrophotometer. The content of flavonoids is expressed in mg rutin equivalent (RE) per g.

### 3.7. Total phenolic contents (TPC)

The total phenolic content was quantified according to Velioglu *et al*. [56]. The Folin-Ciocalteu reagent (diluted 10-fold) was used to determine the total phenolic contents of the samples. One hundred microliters of honey sample (0.1–0.4 g/mL) in distilled water or methanol was mixed with the Folin-Ciocalteu reagent (0.75 mL) and allowed to stand at 22 °C for 5 minutes before adding to sodium bicarbonate solution (0.75 mL, 60 g/L). After 90 minutes at 22 °C, the absorbance was measured at 725 nm. The results were expressed as mg rutin equivalent (RE) per g.

### 3.8. Extraction of phenolic compounds from honey by solid phase extraction (SPE)

The honey extract was prepared as described in previous studies [51] with some modifications. Briefly, C18 SPE cartridges were preconditioned for phenolic compounds by sequentially passing methanol (8 mL) and 0.01 M HCl (4 mL). The honey samples (200 mg) were thoroughly mixed with (1 mL) deionized water for 30 min, until completely dissolved. The resulting honey solution was then filtered under vacuum to remove any solid particles. This solution was adjusted to pH 2.0 with 2M HCl, passed through the preconditioned C18 column and washed with 0.01 M HCl (5 mL). The adsorbed fractions were eluted with methanol (12 mL) and evaporated using a rotary evaporator until dry at 40 °C with a water bath. The residues were re-dissolved in methanol (1 mL) for HPLC measurement; 20 µL of sample was then injected into the HPLC system.

### 3.9. HPLC analysis

Twenty microliters of standard mixtures of gallic acid, chlorogenic acid, caffeic acid, ellagic acid, ferulic acid, *p*-coumaric acid, rutin, hesperetin, qurecetin and chrysin (100 µg/mL for each) and phenolic extracts were injected into the HPLC machine (10A Shimadzu, Japan). The phenolic compounds were detected using UV absorption spectra and monitored at 290 nm and 340 nm; the majority of the honey flavonoids and phenolic acids showed maximum UV absorption at these two wavelengths [57]. The column used was a reversed phase C18 column, ACE (4.6 × 250 mm, particle size 5 µM, USA). The mobile phases were 0.25% formic acid and 2% methanol in water (solvent A) and methanol (solvent B), at constant solvent flow rate of 1 mL/min. The following gradient was used, according to the previously mentioned method [57], except for minor modifications: 10% methanol (B) was flowed through the column isocratically with 90% solvent (A) for 15 min which was then increased to 40% methanol (B) for 20 min, to 45% methanol (B) for 30 min, to 60% methanol (B) for 50 min, to 80% methanol (B) for 52 min, to 90% methanol (B) for 60 min, and then followed by isocratic elution with 90% methanol (B) for 65 min. Finally, the gradient was changed to 10% methanol for 68 min, and this composition was held until 73 min. The phenolic and flavonoid compounds were identified by comparing the chromatographic retention time with those authentic standards. A calibration curve of caffeic acid at 290 nm was used to calculate phenolic acids concentrations, whereas calibration curve of quercetin at 340 nm was used for flavonoids. This is because the different phenolic compounds are absorbed better at these wavelengths [57]. The calibration curves of the standards were used to determine the concentrations of the phenolic compounds in the extracts. 

### 3.10. Statistical analysis

All data were expressed as the mean ± standard deviation (n = 3). The results were analyzed statistically with One-way ANOVA using SPSS version 16.0 software. Differences were considered significant at levels of p < 0.05.

## 4. Conclusions

Our results indicate that the antioxidant activity of Gelam honey was significantly higher than that of Nenas honey, which may be a result of the differences in their phenolic and flavonoid contents. A high correlation was found between the antioxidant activity of honey and its total phenolic content, indicating that the antioxidant activity of honey is attributed by phenolics. This study also emphasized the relevance of honey as a healthy food supplement and as a source of natural antioxidants. Gamma irradiation not only imparts sterility to honey but it also increases the antioxidant capacity of honey due to increased formation of phenolics. Thus, this investigation suggests that radiation treatment at 25 kGy is not only useful in sterilizing the honey but also enhances the antioxidant activity of honey.
